# Diffuse Gastric Cancer During Pregnancy: Report of a Rare Association

**DOI:** 10.14740/wjon945w

**Published:** 2015-10-26

**Authors:** Orivaldo Alves Barbosa, Anna Dorotheia Bezerra de Souza, Mayna Raphaela de Carvalho Moura, Elson Jose de Almeida Junior, Joao Paulo Uchoa Fontenele, Fabio de Figuereido Chaves, Jose Walter Correia

**Affiliations:** a Internal Medicine Department, Hospital Cesar Cals, Brazil; b Department of Gynecology and Obstetrics, Hospital Cesar Cals, Brazil; c Laboratory Argos, Brazil; d Ceara Cancer Institute, Brazil

**Keywords:** Gastric cancer, Pregnancy, Peritoneal metastases, Prognosis, Prematurity

## Abstract

Gastric cancer during pregnancy is a rare event and difficult to diagnose, as the symptoms can be confused with those of an ordinary pregnancy. We report a case of a 25-year-old patient with a 29-week gestation, with asthenia complaint, vomiting and weight loss. During the investigation of wasting syndrome endoscopy was performed with infiltrative ulcerative lesions in pre-pyloric region with biopsy revealing carcinoma with signet ring, undifferentiated type. It was held on a strict control of fetal vitality, and pregnancy was interrupted via the abdominal delivery at 34 weeks. Soon after the cesarean section was performed, exploratory laparotomy was performed to perform inventory of the abdominal cavity, being observed the presence of carcinomatous implants in the peritoneum. In the face of irresectability clinical conduct was adopted and the patient was sent to chemotherapy, ensuring nutrition via a jejunostomy. The article reviews the gastric carcinoma association with pregnancy, discussing the initiation of treatment and continuity of pregnancy.

## Introduction

Gastric carcinoma is extremely rare during pregnancy, usually having late diagnosis in advanced stages, with a bad maternal and fetal prognosis. Symptoms of cancer can be confused with other disorders of the period, as hyperemesis gravidarum, hindering early diagnosis of these cases [1].

We report a case of a young pregnant patient with late diagnosis of gastric diffuse type carcinoma.

## Case Report

The study was approved by the Research Ethics Committee of the Hospital Cesar Cals, Fortaleza, Ceara. The patient consented to the story, according to CNS Resolution 196/96.

A female patient, 25, with a preterm labor in previous pregnancy, was admitted to the hospital with a gestational age of 29 weeks and 6 days with complaints of asthenia, vomiting (3 - 4 episodes/day) and hair loss especially 1 week prior to admission. She reports also weight loss, approximately 14 kg in 6 months. She refers treatment for chronic gastritis with ranitidine 150 mg/day since July 2014. The obstetric ultrasound performed on admission showed only pregnancy, longitudinal, cephalic, with fetal weight 1,083 g (between third and 10th percentiles), amniotic fluid index 88 mm, anterior placenta and normal body Doppler velocimetry of the fetal-placental circulation.

**Figure 1:**
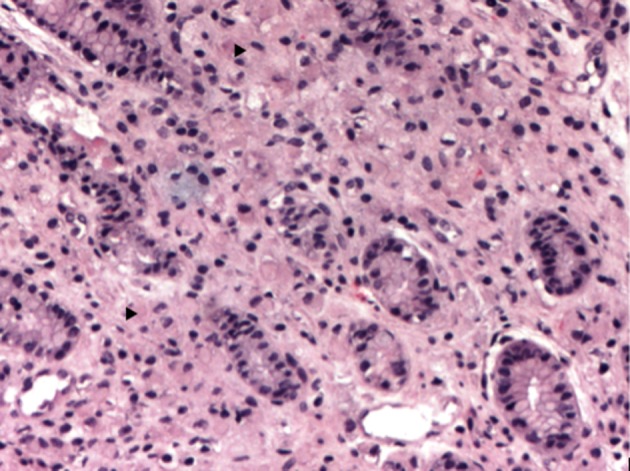
Multiple small cells infiltrating the lamina propria of the gastric mucosa, H&E, × 100 (arrow).

**Figure 2:**
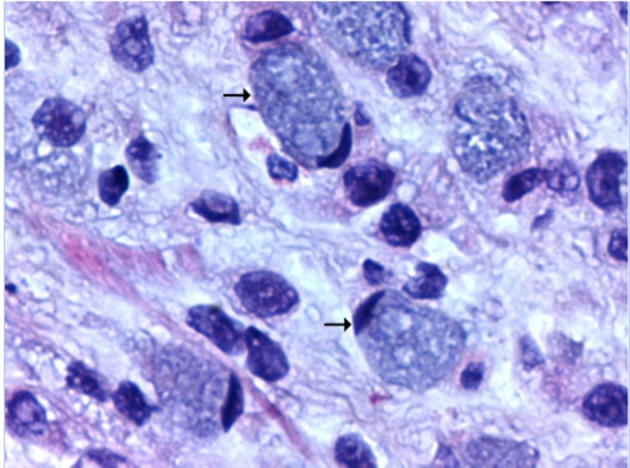
Detail of signet ring cell with mucinous cytoplasm and core diverted to the cell periphery, H&E, × 400 (arrow).

**Figure 3:**
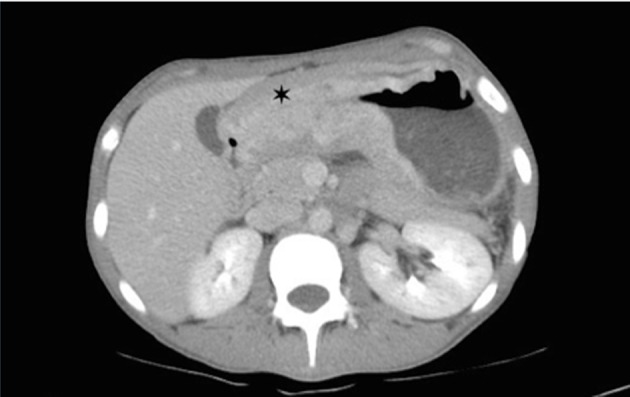
Abdominal tomography with intravenous contrast revealing intense thickening of the gastric wall with diffuse narrowing in the pre-pyloric region (asterisk).

Corticosteroids were prescribed for fetal lung maturity and investigation started for wasting syndrome. In upper GI endoscopy a tumor lesion was observed invading the whole gastric antrum (infiltrative aspect Borrmann IV) not being possible to guide nasogastric tube, biopsies were made and parenteral nutrition started. The histopathological study was compatible with signet ring carcinoma, undifferentiated Nakamura or diffuses Lauren (Fig. 1, 2) negative for HER2 receptor and estrogen receptor. The patient chose to wait until 34 weeks for resolution of pregnancy, abdominal delivery being held without complications and in a second surgical time, laparotomy revealing suggestive peritoneal carcinomatosis, confirmed by histopathology. The baby was a female, weighed 1,400 g and had Apgar 7:8 in the first and fifth min respectively, without mechanical ventilation requirement. Chest and abdomen postoperative CT scans were performed (Fig. 3) where locally advanced disease was observed. The postpartum evolved without surgical complications and was referred to oncology service in order to perform chemotherapy, being initiated scheme with cisplatin and capecitabine, with good tolerance for the patient with a partial response in 4 months after initiation of therapy.

## Discussion

Gastric cancer during pregnancy is considered a rare event, representing approximately 0.1% of stomach tumors, with about 136 reported cases in literature, especially in Japan [2].

Often such patients have belatedly diagnosed malignancies. According to Sakamoto et al [3], 92.5% of diagnosed patients possessed advanced gastric cancer and ominous prognosis; survival at 1 and 2 years was 18.0% and 15.1%, respectively, with a delay in diagnosis and worse survival for several reasons. 1) Symptoms of gastric cancer are similar to common symptoms during this period, such as vomiting, dyspepsia and increased abdominal size. 2) The estrogen hormone environment appears to favor the growth of neoplastic cells, as evidenced in studies with positive estrogen receptor in 55.8% of gastric tumors [4]. 3) The presence of *Helicobacter pylori* is prevalent in pregnant women [5]. 4) The spread of cancer can be facilitated by the increased circulatory flow that occurs during pregnancy.

Upper digestive endoscopy, method of choice for diagnosis in these cases, is considered low-risk procedure during pregnancy and should not be delayed with good clinical indication [6]; if possible, should be performed in the second trimester of pregnancy.

The handling of such cases remains a challenge because of the conflicting needs of immediate treatment and continuation of pregnancy. A treatment plan that includes a multidisciplinary team of obstetricians, surgeons and oncologists should consider gestational age and decide the best therapeutic approach. Generally, chemotherapeutic treatments are considered safe after the first trimester, and should be avoided 3 weeks before the resolution time because of the risk of hematological toxicity and fetal immunosuppression. In most cases, tumors diagnosed before the 20th week of pregnancy are conducted with therapeutic abortion and after the third quarters should be opted for early termination of pregnancy, but the discussion with the multidisciplinary team, the patient and family is essential in the decision [7]. In our case, the patient chose to wait until the 34th week of pregnancy, in order to reduce fetal morbidity with a good outcome for both the mother and the baby.

### Conclusion

Gastric cancer in pregnancy, although extremely rare, should be considered in the differential diagnosis of wasting syndrome and repetitive vomiting, especially when there are risk factors.
